# Rationale and design of decision: a double-blind, randomized, placebo-controlled phase III trial evaluating the efficacy and safety of sorafenib in patients with locally advanced or metastatic radioactive iodine (RAI)-refractory, differentiated thyroid cancer

**DOI:** 10.1186/1471-2407-11-349

**Published:** 2011-08-11

**Authors:** Marcia S Brose, Christopher M Nutting, Steven I Sherman, Young Kee Shong, Johannes WA Smit, Gerhard Reike, John Chung, Joachim Kalmus, Christian Kappeler, Martin Schlumberger

**Affiliations:** 1 Department of Otorhinolaryngology: Head and Neck Surgery, Department of Medicine, Division of Hematology/Oncology, The University of Pennsylvania, Abramson Cancer Center, Clinical Research Building, Room 127, 425 Curie Boulevard, Philadelphia, PA 19104 USA; 2 Head and Neck Unit, Royal Marsden Hospital, Fulham Road, London, SW3 6JJ, UK; 3 Department of Endocrine Neoplasia and Hormonal Disorders, The University of Texas MD Anderson Cancer Center, Endocrine Multidisciplinary Center, Unit 1461, 1400 Pressler Street, Houston, TX 77230-1402 USA; 4 Department of Internal Medicine, Asian Medical Center, University of Ulsan College of Medicine, 388-1 Pungnap-dong, Songpa-gu, Seoul 138-736, Korea; 5 Department of Medicine, Endocrinology, and Metabolic Diseases, Leiden University Medical Center, Albinusdreef 2, 2333 ZA Leiden, The Netherlands; 6 Bayer Schering Pharma AG, Mullerstrasse 178, 13353 Berlin, Germany; 7 US Medical Sciences, Oncology, Bayer HealthCare Pharmaceuticals, 6 West Belt, Wayne, NJ 07470 USA; 8 Institut Gustave Roussy, rue Camille Desmoulins, 94805 Villejuif Cédex, France

## Abstract

**Background:**

The incidence of thyroid cancer and the number of patients who die from this disease are increasing globally. Differentiated thyroid cancer (DTC) is the histologic subtype present in most patients and is primarily responsible for the increased overall incidence of thyroid cancer. Sorafenib is a multikinase inhibitor that targets several molecular signals believed to be involved in the pathogenesis of thyroid cancer, including those implicated in DTC. In phase II studies of patients with DTC, sorafenib treatment has yielded a median progression-free survival (PFS) of 58 to 84 weeks and disease control rates of 59% to 100%. The DECISION trial was designed to assess the ability of sorafenib to improve PFS in patients with locally advanced or metastatic, radioactive iodine (RAI)-refractory DTC.

**Methods/design:**

DECISION is a multicenter, double-blind, randomized, placebo-controlled phase III study in patients with locally advanced/metastatic RAI**-**refractory DTC. Study treatment will continue until radiographically documented disease progression, unacceptable toxicity, noncompliance, or withdrawal of consent. Efficacy will be evaluated every 56 days (2 cycles), whereas safety will be evaluated every 28 days (1 cycle) for the first 8 months and every 56 days thereafter. Following disease progression, patients may continue or start sorafenib, depending on whether they were randomized to receive sorafenib or placebo, at investigator discretion. Patients originally randomized to receive sorafenib will be followed up every 3 months for overall survival (OS); patients originally randomized to receive placebo will be followed up every month for 8 months after cross-over to sorafenib. The duration of the trial is expected to be 30 months from the time the first patient is randomized until the planned number of PFS events is attained. The primary endpoint is PFS; secondary endpoints include OS, time to disease progression, disease control rate, response rate, duration of response, safety, and pharmacokinetic analysis.

**Discussion:**

The DECISION study has been designed to test whether sorafenib improves PFS in patients with locally advanced or metastatic RAI-refractory DTC.

**Trial Registration:**

ClinicalTrials.gov Identifier: NCT00984282; EudraCT: 2009-012007-25.

## Background

In the United States in 2010, there were 44,670 estimated new cases of thyroid cancer, making it the most common of the endocrine malignancies [1]. The incidence of thyroid cancer is increasing globally, as is the number of patients who die from this disease [2,3]. Differentiated thyroid cancer (DTC) is the histologic subtype present in most patients, as well as being primarily responsible for the increased overall incidence of thyroid cancer [4].

Therapeutic options for patients with radioiodine (RAI)-refractory, advanced DTC are limited. The only systemic therapy approved in most countries is doxorubicin, which has traditionally been a limited option because complete responses are rare, partial responses are limited, and toxicity is considerable [5]. DTC is therefore a disease in which new therapeutic options with favorable toxicity profiles are greatly needed.

**Figure 1:**
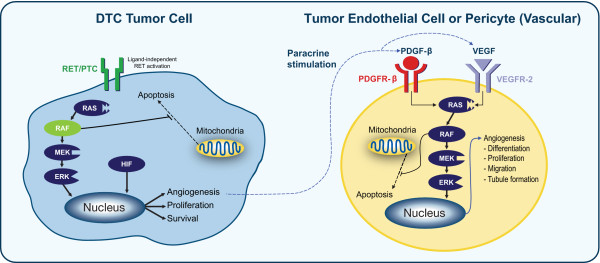
**Molecular signals believed to be involved in the pathogenesis of DTC**. Key events in the initiation of thyroid cancer are believed to include constitutive activation of BRAF, RAS, and RET/PTC [17,18].

Sorafenib is a multikinase inhibitor that targets several molecular signals believed to be involved in the pathogenesis of thyroid cancer, including those implicated in DTC. These signals include the RAS and BRAF/MEK/ERK signaling pathways; ligand-independent RET/PTC receptor tyrosine kinase activation; and pathways involving vascular endothelial growth factor (VEGF), platelet-derived growth factor (PDGF), and their receptors (Figure 1).

A total of 168 patients with thyroid cancer, including 133 (79%) with differentiated histology, have been treated with sorafenib in four phase II trials (Table 1). Median progression-free survival (PFS) ranged from 58 weeks to 84 weeks, partial responses (PRs) were observed in up to 25% of patients, and disease control rate (DCR [stable disease + partial response]) ranged from 59% to 100%. These results were achieved despite dose reductions due to adverse events (AEs) in up to 62% of patients. Most AEs experienced by patients in these phase II trials were grade 1 or 2 and were manageable.

**Table 1:** Phase II trials of sorafenib in DTC

Trial	Efficacy Results	Grade ≥ 3 AEs in ≥ 5% of Patients	Other
Single-arm trial (N = 55 total; n = 47 DTC) [10-12]	mPFS = 84 weeks* SD = 53.3%^†^ PR = 23.3%^†^ DCR = 76.6%^†^ OS = 140 weeks^†^	Hypertension (13%), hand-foot syndrome (10%), rash (10%), weight loss (10%), diarrhea (7%), elevated LFTs (7%)^‡^	• Dose reduction (due to AEs) was required in 47% of patients (initial analysis) • Median duration of treatment at initial analysis (30 of 55 patients accrued) = 27 weeks • BRAF genotyping (n = 16): mPFS = 84+ weeks in DTC patients with BRAF^V600E^, compared with 54 weeks in those with BRAF^wt ^(*P *= 0.028)
Single-arm component (chemotherapy-naive patients with DTC) of two-arm trial (N = 56 total; n = 41 DTC) [13]	mPFS = 65 weeks* SD = 57%* PR = 15%* DCR = 72%*	Fatigue, HSFR (11% each); weight loss, skin rash, hypertension, diarrhea, stomatitis, tongue/tooth pain, abdominal/rectal pain, proximal myopathy, back pain, general pain, hand/foot pain, arthralgia, colon perforation (5% each)	• Dose reduction (due to AEs) was required in 52% of patients
Single-arm trial (N = 26 total; n = 14 DTC) [14,15]	mPFS not reported SD = 82%^†^ PR = 18%^†^ DCR = 100%^†^	Hand-foot syndrome (19%), other skin toxicity (6%), hypertension (6%), infection (8%)	• Dose reduction (due to AEs) was required in 62% of patients • Five drug-related serious AEs were reported: 2 hospitalizations for non-neutropenic fever/infection, 1 for hypocalcemia, and 2 for fever/rash
Single-arm trial (N = 31; all DTC) [16]	mPFS = 58 weeks SD = 34% PR = 25% DCR = 59%	HFSR (22%), hypertension (16%), weight loss (9%)	• Dose reduction (due to AEs) was required in 56% of patients

*DTC cohort

^†^Total study sample

^‡^One patient developed grade 3/4 LFT abnormalities at 8 weeks and died of liver failure 12 weeks later

DTC = differentiated thyroid cancer; mPFS = median progression-free survival; SD = stable disease; PR = partial response;

DCR = disease control rate; LFTs = liver function tests; HFSR = hand-foot skin reaction; AE = adverse event

### Rationale for phase III study

Given the encouraging results from phase II trials of sorafenib in patients with DTC and the need for improvement in the treatment of thyroid cancer, the phase III DECISION (Study of Sorafenib in Locally Advanced or Metastatic Patients with RAI-Refractory Thyroid Cancer) trial was initiated to evaluate the efficacy and safety of sorafenib in patients with locally advanced or metastatic, RAI-refractory, DTC. Because PFS may better predict improvement in overall survival (OS) than response rate, PFS was selected as the primary efficacy assessment.

## Methods/design

### Primary Objective

Patients with RAI-refractory DTC (papillary, follicular, Hürthle cell, and poorly differentiated carcinoma) are being randomized to receive sorafenib or placebo. The primary objective of this phase III study is to compare PFS, as evaluated by Response Evaluation Criteria in Solid Tumors version 1.0 (RECIST)[6], in the sorafenib and placebo groups. PFS is defined as the time from date of randomization to the date of radiologic progression or death (if death occurs before progression).

### Secondary Objectives

Among the secondary efficacy objectives of this trial are OS, measured from date of randomization to date of death due to any cause; time to progression (TTP), measured from date of randomization to date of confirmed radiologic progression; and disease control rate (DCR), defined as the proportion of patients with a best overall tumor response of complete response (CR), PR, or stable disease (SD) based on RECIST criteria, during treatment or within 30 days of termination of study medication. Other secondary efficacy objectives include response rate (RR), defined as the proportion of patients with a best overall tumor response of PR or CR during treatment or within 30 days of termination of study medication; and duration of response (DoR), defined as the time from first documented objective response of PR or CR (whichever is noted earlier) to disease progression or death (if death occurs before progression is documented). Secondary safety objectives include assessment of AEs and abnormalities in laboratory parameters. Pharmacokinetic (PK) objectives include determinations of exposure to sorafenib, defined as the area under the concentration (AUC) time curve from time 0 to 12 hours (AUC_0-12_) by population PK methods and using previously developed sorafenib population PK models.

### Design/Randomization

**Figure 2:**
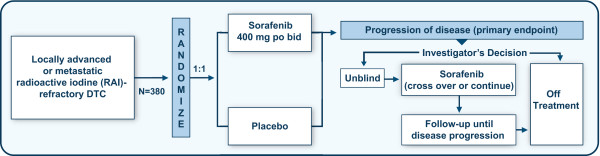
**DECISION schema**.

DECISION is a randomized, double-blind, placebo-controlled, phase III trial, in which approximately 380 patients will be randomized 1:1 to receive sorafenib or placebo (Figure 2).

### Stratification

Patients are being stratified at randomization according to age (< 60 vs ≥ 60 years) and geographic region (North America vs Europe vs Asia).

### Setting

The study setting is multinational, with approximately 91 sites in North America, Europe, and Asia. Patients are currently being enrolled in 19 countries: the United States, Germany, Italy, France, Poland, UK, Denmark, Spain, Sweden, Netherlands, Austria, Belgium, China, South Korea, Japan, Russia, Slovakia, Bulgaria, and Saudi Arabia.

### Ethics, Informed Consent, and Safety

Documented approval has been obtained from appropriate Ethics Committees/Institutional Review Boards at all participating centers/countries prior to study start. The study is also designed to conform to International Conference on Harmonization of good clinical practice (GCP) guidelines, local laws, regulations, and organizations, as well as with the Declaration of Helsinki. Written informed consent must be obtained from each patient before any study-specific procedure takes place. Participation in the study and date of informed consent patient are being documented appropriately in each patient's files. A Data Monitoring Committee is in place to monitor the trial.

### Eligibility

#### Inclusion Criteria

All patients must be > 18 years of age, have a life expectancy of at least 12 weeks, and have locally advanced or metastatic DTC (papillary, follicular, Hürthle cell, or poorly differentiated carcinoma) with at least one measurable lesion as measured by computed tomography (CT) or magnetic resonance imaging (MRI) and disease progression within 14 months. Patients must not be candidates for curative surgery or radiation therapy. Additional inclusion criteria are an Eastern Cooperative Oncology Group (ECOG) performance status ≤ 2 [7]; adequate thyroid-stimulating hormone (TSH) suppression (< 0.5 mU/L); and adequate bone marrow, liver, and renal function. All patients must have RAI-refractory disease, defined as a target lesion with no iodine uptake on a post-RAI scan performed under conditions of a low iodine diet and adequate TSH elevation or recombinant human TSH (rhTSH) stimulation. However, certain patients who have had some iodine uptake may also be eligible. They include: (1) patients who have undergone a single RAI treatment (≥ 3.7 GBq [≥ 100 mCi]) within the previous 16 months, and who have progression of the target lesion despite RAI treatment; (2) patients who have had multiple RAI treatments, with the last RAI treatment > 16 months ago, and who had disease progression after each of two RAI treatments (≥ 3.7 GBq [≥ 100 mCi] each) administered within 16 months of each other; and (3) those who have received a cumulative RAI dose of ≥ 22.2 GBq (≥ 600 mCi).

#### Exclusion Criteria

Patients who undergo major surgery, open biopsy, or significant traumatic injury ≤ 30 days prior to randomization; those with previous or concurrent cancer distinct in primary site or histology from thyroid cancer ≤ 5 years prior to randomization, except for cervical cancer in situ, treated basal-cell carcinoma, and superficial (Ta, Tis, or T1) bladder tumors; and patients with foci of undifferentiated thyroid cancer are being excluded from the study. Other exclusion criteria include the presence of a non-healing wound, ulcer, bone fracture, or grade ≥ 2 infection according to National Cancer Institute Common Terminology Criteria for Adverse Events (NCI-CTCAE) v3.0 [8], a grade ≥ 3 hemorrhage or bleeding event according to NCI-CTCAE ≤ 3 months prior to randomization; evidence or history of bleeding diathesis or coagulopathy; or the presence of tracheal, bronchial, or esophageal infiltration with significant risk of bleeding (but without having received local treatment prior to enrollment in the study). Patients with clinically significant cardiac disease and/or uncontrolled hypertension (> 150/90 mm Hg) despite optimal treatment; those known to be infected with human immunodeficiency virus (HIV) or hepatitis B (HBV) or C (HCV) virus; women who are pregnant or breastfeeding; and patients with a known or suspected allergy to sorafenib or hypersensitivity to sorafenib or any agent given during the course of the study are also being excluded.

#### Excluded Therapies and Medications

Patients are being excluded if they had been treated for cancer with any licensed or investigational tyrosine kinase inhibitors; monoclonal antibodies that target vascular endothelial growth factor (VEGF), VEGF receptors, or other targeted agents; cytotoxic chemotherapy agents (except for prior low-dose chemotherapy for radiosensitization); or thalidomide or any of its derivatives. Enrolled patients are unable to receive concomitant RAI, chemotherapy, or other investigational therapy; or any substances known to induce CYP3A4 (eg, St. John's Wort, dexamethasone > 16 mg daily, phenytoin, carbamazepine, rifampin, rifabutin, phenobarbital) within 7 days of randomization.

#### Concomitant Medication Precautions

Patients taking medications with a narrow therapeutic index (eg, warfarin) are being proactively monitored. In addition, because sorafenib inhibits a variety of liver metabolic enzymes in vitro, patients taking concomitant medications known to be metabolized by the liver are being monitored closely for AEs associated with those medications, especially as the clinical effects of sorafenib in patients taking drugs metabolized by these enzymes are unknown.

### Estimated Timeline

The expected study duration is 30 months. The study will be halted when data for the primary endpoint are mature. Any patients continuing to benefit from treatment at the time of the study endpoint will be allowed to continue treatment.

### Treatments

Patients are being randomized to receive sorafenib 400 mg or matching placebo (2 tablets), twice daily (approximately every 12 hours apart without food; at least 1 hour before or 2 hours after a meal) in a double-blind fashion; neither the investigator nor the patient (or sponsor) will know which agent is being administered. Treatment will be continued until radiographically documented disease progression, unacceptable toxicity, or the study endpoint. Sorafenib will be made available, via an extension program or other mechanism, to patients who continue to show benefit after the study endpoint, until disease progression or unacceptable toxicity.

In the event of confirmed radiologic progression, as determined by RECIST v1.0 criteria, study treatment may be unblinded. Patients who had been randomized to receive sorafenib may continue to receive sorafenib, whereas those randomized to receive placebo may cross over to sorafenib. Decisions about continuing study medication and cross-over will be made by the investigator, based on each patient's clinical status and the determination of the investigator that the patient may receive clinical benefit from sorafenib.

### Safety Assessments

**Figure 3:**
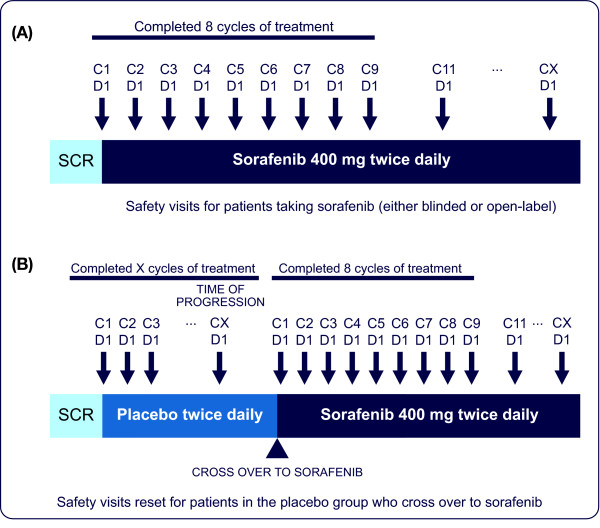
**Safety assessments**. (A) Patients treated with sorafenib during the double-blind treatment period and those treated with sorafenib who experience PD before completing 8 cycles and are unblinded (open-label treatment period). (B) Patients who received placebo during the double-blind treatment period and then crossed over to sorafenib after unblinding.

Patients are being assessed for safety every 28 days (1 cycle) for the first 8 months and every 56 days (2 cycles) thereafter (Figure 3). Patients are being monitored for AEs using NCI-CTCAE v3.0 criteria. Treatment-emergent AEs and safety laboratory parameters will be summarized by treatment group.

### Efficacy Assessments

Efficacy is being evaluated every 56 days. Following progressive disease and at the discretion of the investigator, patients may continue or start sorafenib, depending on the treatment arm. Patients treated after progressive disease will be followed for efficacy until further progressive disease and for safety until 30 days after the end of sorafenib treatment. Dose modifications or interruptions are being allowed, based on specific criteria, for grade 2-3 hand-foot skin reaction (HFSR) and other AEs. Patients will enter the long-term follow-up period upon discontinuation of randomized therapy (sorafenib or placebo), based on documentation of progressive disease or unacceptable toxicity.

### Statistical Analysis

The primary population for efficacy analysis will be the intention-to-treat (ITT) population, defined as all randomized patients. The population for safety analysis will consist of all patients who receive at least one dose of study medication. All randomized patients (ITT population) will be included in the primary analysis. The primary efficacy endpoint is PFS, with analysis performed when a predetermined number of PFS events has occurred. The null hypothesis--that both treatment arms have the same PFS distribution--will be tested against the alternative hypothesis--that the distribution of PFS in the two arms will differ according to Lehmann's alternative.

The two treatment groups (sorafenib and placebo) will be compared using a one-sided stratified log-rank test, with an overall one-sided alpha of 0.01 stratified by age (< 60 years vs ≥ 60 years) and geographica region (North America, Europe, and Asia). Kaplan-Meier estimates of median times to PFS and Kaplan-Meier curves will be generated for each group. The hazard ratios and 95% confidence intervals will be derived from a Cox proportional hazards model.

The secondary efficacy endpoints of TTP and OS in each group will be analyzed by the Kaplan-Meier method and compared using the log-rank test. Response rate and DCR will be analyzed using the Cochran-Mantel-Haenszel test. The tests will be adjusted for the same stratification factors as used for PFS. Duration of response and the exploratory endpoints will be analyzed with descriptive statistics only.

## Discussion

Currently, there is no standard of care for the treatment of patients with advanced, RAI-refractory DTC [9]. In designing the randomized, placebo-controlled DECISION study, we hypothesized that the inclusion of a cross-over component would likely be the best approach to recruit and retain patients in this study. Although this design precludes an endpoint such as OS, patients with advanced DTC may derive benefit from a well-tolerated therapy such as sorafenib. Therefore, DECISION is designed to demonstrate PFS when patients with locally advanced or metastatic RAI-refractory DTC are treated with sorafenib.

## Competing interests

Dr. **Marcia S. Brose **has received grant/research support from Onyx, Bayer HealthCare, Novartis, Daiichi-Sankyo, Exelixis and Plexicon/Roche/Genetech; has received honoraria from Onyx and Bayer HealthCare; and is a consultant for AstraZeneca and Bayer HealthCare.

Dr. **Martin Schlumberger **has participated on advisory boards and received research grants from AstraZeneca, Bayer HealthCare, Exelixis, Eisai, and Genzyme.

Dr. **Christopher M. Nutting **has received grant/research support from Bayer HealthCare.

Dr. **Steven I. Sherman **has received grant/research support from Amgen, AstraZeneca, Eisai, and Genzyme; has been a consultant for AstraZeneca, Bayer HealthCare, Exelixis, Plexxikon, Semafore, Oxigene, and Eli Lilly; and has received honoraria from Genzyme and Exelixis.

Dr. **Young Kee Shong **is a consultant for Bayer HealthCare.

Dr. **Johannes W.A. Smit **has nothing to disclose.

Drs. **Gerhard Reike**, **John Chung**, **Joachim Kalmus **and **Christian Kappeler **are employees of Bayer HealthCare.

## Authors' contributions

MSB and MS, the principal investigators on the DECISION trial, supervised the study and acquired the data; were extensively involved with the DECISION study concept and design; and were involved in drafting and critically revising the manuscript for intellectual content. CMN, SIS, YKS, and JWAS, co-investigators on the DECISION trial, also supervised the study and acquired the data; were extensively involved with the DECISION study concept and design; and were involved in drafting and critically revising the manuscript for intellectual content. GR, JC, JK, and CK assisted in supervising the study, acquiring the data, and providing administrative and technical support as well as were extensively involved with the DECISION study concept and design and in drafting and critically revising the manuscript for intellectual content. All authors read and approved the final manuscript.

## Pre-publication history

The pre-publication history for this paper can be accessed here:

http://www.biomedcentral.com/1471-2407/11/349/prepub
